# Supplementary material for: Superionic effect and anisotropic texture in Earth’s inner core driven by geomagnetic field
Source: Nat Commun. 2023 Mar 24;14:1656. doi: 10.1038/s41467-023-37376-1 (PMC10039083; doi:10.1038/s41467-023-37376-1)
Supplement: Supplementary file 1 — Supplementary Information [file 41467_2023_37376_MOESM1_ESM.pdf]

Supplementary Information

Superionic effect and anisotropic texture in Earth's inner core driven by geomagnetic field

Shichuan Sun[1,2], Yu He[1,2,3,*], Junyi Yang[1,2], Yufeng Lin[4], Jinfeng Li[4], Duck Young Kim[3], Heping Li[1,2], Ho-kwang Mao[3]

[1]Key Laboratory of High-Temperature and High-Pressure Study of the Earth's Interior, Institute of Geochemistry, Chinese Academy of Sciences, Guiyang 550081, Guizhou, China

[2]University of Chinese Academy of Sciences, Beijing 100049, China

[3]Center for High Pressure Science and Technology Advanced Research, Shanghai 201203, China

[4]Department of Earth and Space Sciences, Southern University of Science and Technology, Shenzhen 518055, China

*E-mail: heyu@mail.gyig.ac.cn

Supplementary Discussion 1. Seismic velocities in superionic Fe-H alloys under inner core conditions

Supplementary Discussion 2. Reversal of the fast velocity axis and change in the c/a ratio in superionic Fe-H alloys

Supplementary Discussion 3. Elastic anisotropy in Fe-H and other Fe alloys

Supplementary Discussion 4. Comparison of Fe-H seismic anisotropy with seismological observations

Supplementary Discussion 5. H-ion anisotropic diffusion in Fe-H alloys

Supplementary Discussion 6. H-ion transportation in an external electric field

Supplementary Discussion 7. Bader charge analysis of Fe alloys

Supplementary References

Supplementary Discussion 1. Seismic velocities in superionic Fe-H alloys under inner core conditions.

In this section, we provide a detailed calculation method and data for the elasticity and seismic velocities of superionic Fe-H alloys.

The elastic properties of Fe-H alloys are calculated under various temperatures (2000-6000 K), pressures (330-360 GPa) and H contents (0.0625, 0.125 and 0.25 atomic percent) using the AIMD method. This method has been used for elastic property calculations in our previous studies[1-3]. The calculated elastic constants show good consistency with the experimental results of the superionic material Li_2O at high temperatures[3]. The elastic properties of crystals are expressed as the relationship between stress and strain:

$$\sigma_{ij} = C_{ijkl}\varepsilon_{kl}, \tag{1}$$

where σ_{ij} refers to a stress tensor, ε_{kl} refers to a strain tensor, and C_{ijkl} represents a fourth-order elastic modulus. The elastic constant C_{ij} was calculated by distorting the equilibrium structure and solving the stress–strain relations (see method for details; Table 1).

The statistical errors of the calculated temperature and stress were evaluated using the blocking method[4]. The standard deviation is defined as follows[5]:

$$\delta \approx \sqrt{\frac{C_0'}{n'-1}\left(1+\frac{1}{\sqrt{2(n'-1)}}\right)} \tag{2}$$

$$n' = \frac{n}{2} \tag{3}$$

$$x_i' = \frac{1}{2}(x_{2i-1} - x_{2i}) \tag{4}$$

$$c_o' = \frac{1}{n'}\sum_{k=1}^{n'}(x_k - \bar{x})^2 \tag{5}$$

where *n* is the number of data points. The standard deviations of the time-averaged properties in the NVT simulations are counted, and the errors are 3-9 K in temperature and 0.02-0.15 GPa in

stress. The errors are ~2% in C_{ij}.

The adiabatic elastic constants (C_{ij}^{S}) were estimated from isothermal elastic constants (C_{ij}^{T}) using the following equations (Table 2)[5,6]:

$$C_{11}^{S} = C_{11}^{T} + \alpha\gamma T K_T \tag{6}$$

$$C_{12}^{S} = C_{12}^{T} + \alpha\gamma T K_T \tag{7}$$

$$C_{13}^{S} = C_{13}^{T} + \alpha\gamma T K_T \tag{8}$$

$$C_{33}^{S} = C_{33}^{T} + \alpha\gamma T K_T \tag{9}$$

$$C_{44}^{S} = C_{44}^{T} \tag{10}$$

$$C_{66}^{S} = C_{66}^{T} \tag{11}$$

$$K_S = K_T(1 + \alpha\gamma T) \tag{12}$$

where α and γ denote the thermal expansion coefficient and Grüneisen parameter ($\alpha = 1 \times 10^{-5}\ K^{-1}$, γ=1.5)[7,8], respectively. T is the temperature, and K_T is the isothermal bulk modulus. It is worth noting that the thermodynamic properties for superionic Fe alloys is not well constrained; thus, the estimated adiabatic elastic constants may present some deviation. In addition, as the thermal conductivity is high in the inner core, the presence of thermal convection, and possible Joule heating due to the penetration of the outer core magnetic field, it is still not clear if the seismic wave velocities in the inner core are isothermal, intermediate or adiabatic. In this case, we still use the isothermal data for discussion. The adiabatic compressional velocity is higher than the isothermal velocity, but the difference in compressional velocity anisotropy is negligible (Supplementary Fig. 1 and Supplementary Table 2).

We calculated the bulk modulus B and shear modulus G using the Voigt average scheme, which is proven to be more appropriate and accurate in calculating the seismic wave properties[9].

The compressional wave velocity V_P, shear wave velocity V_S, and bulk sound velocity V_Φ are calculated by:

$$V_P = \sqrt{\frac{B+\frac{4G}{3}}{\rho}}, \quad V_S = \sqrt{\frac{G}{\rho}}, \quad V_\Phi = \sqrt{\frac{B}{\rho}}. \qquad (13)$$

The results are provided in Table S1. We also compared the compressional velocities in superionic FeH alloys with the PREM data[10], and the temperature, pressure and H content effects on velocities and densities are evaluated in Supplementary Fig. 2. Both the densities and velocities of Fe-H alloys decrease with temperature and H content. Increasing the temperature leads to a significant decrease in velocity, while increasing the H content causes a significant reduction in density. Moreover, both the densities and velocities decrease with increasing pressure. The V_P in Fe-H alloys is able to fit the PREM data[10] considering the variation in temperature, pressure and H content in the IC. A higher H content is needed to fit the V_P of the outer inner core (OIC) due to the density deficiency. This may suggest a variation in H content with depth in the IC. The shear wave velocities in Fe-H alloys are still faster than the PREM data (Supplementary Fig. 2b). This suggests the possible existence of other light elements in the IC. In particular, light elements that are more stable at substitutional sites should be considered.

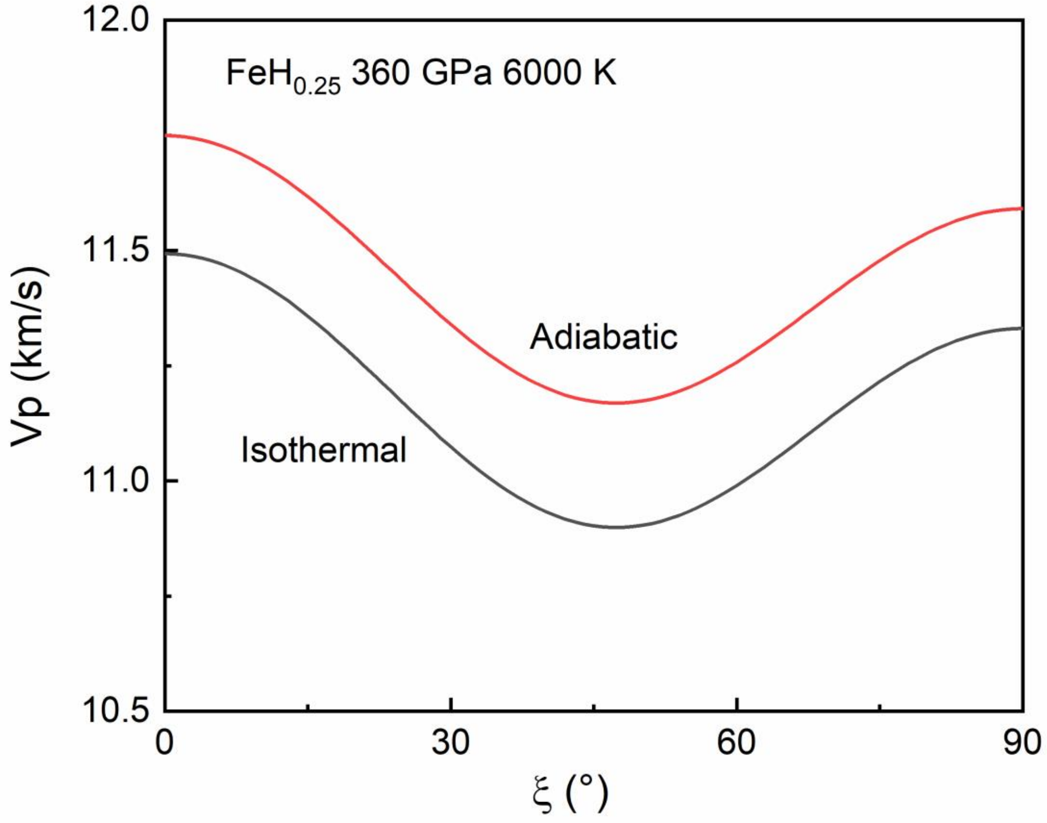

Supplementary Fig. 1 | Adiabatic and isothermal compressional velocity anisotropy in $FeH_{0.25}$ as a function of angle ξ between the ray path and Earth's rotation axis.

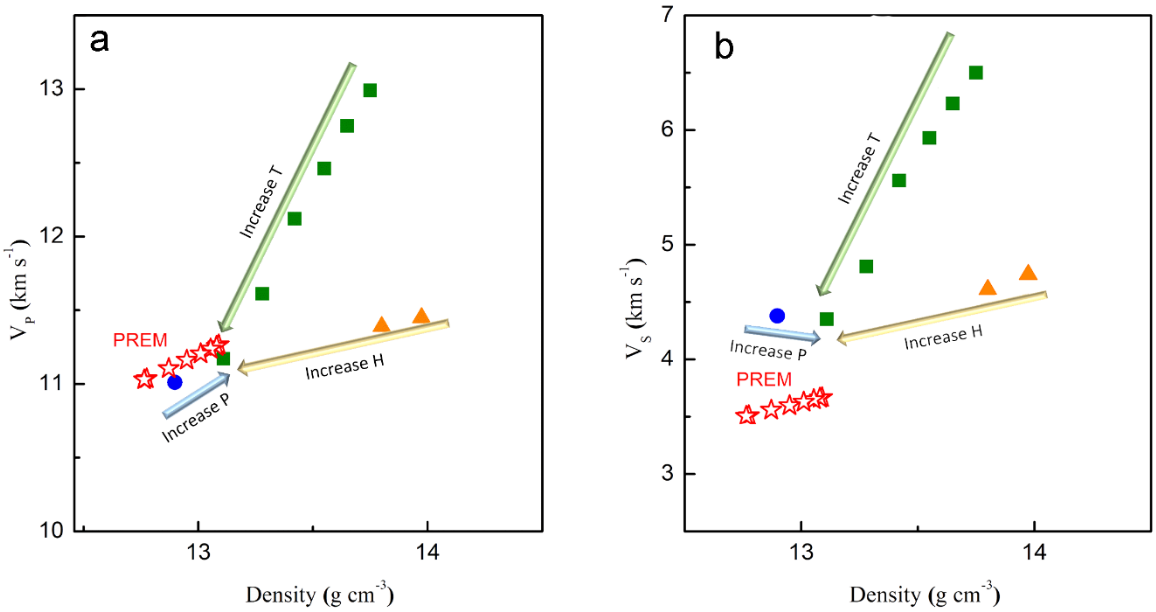

Supplementary Fig. 2 | Calculated seismic velocities in Fe-H alloys in comparison with the PREM data. (**a**) Compressional wave velocities (V_P) and (**b**) shear wave velocities (V_S) in $FeH_{0.25}$ at temperatures from 0 to 6000 K and 360 GPa are shown with green symbols. The V_P and V_S in $FeH_{0.25}$ at 5500 K and 330 GPa are shown with blue symbols. V_P and V_S in $FeH_{0.0625}$ and $FeH_{0.125}$ at 360 GPa and 6000 K are shown with orange symbols. The trends of increasing temperature, pressure and H content are shown with light green, light blue, and yellow arrows.

Supplementary Table 1. The isothermal elastic properties of Fe alloys under inner core conditions

	P (GPa)	T (K)	ρ (g cm^{-3})	C_{11} (GPa)	C_{12} (GPa)	C_{33} (GPa)	C_{13} (GPa)	C_{44} (GPa)	AV_P (%)	AV_S (%)
$FeH_{0.25}$	360	0	13.751	2390	1202	2529	1107	508	5.1	10.4
	360	2000	13.654	2273	1178	2373	1085	458	4.7	10.6
	360	3000	13.552	2195	1184	2195	1070	394	4.0	12.3
	360	4000	13.423	2050	1158	2059	1073	340	3.9	13.6
	360	5000	13.277	1869	1214	1830	1111	248	4.0	13.9
	330	5500	12.898	1648	1165	1613	968	198	4.8	20.7
	360	6000	13.118	1733	1178	1684	1054	178	5.3	22.1
$FeH_{0.0625}$	360	6000	13.973	1894	1270	1925	1115	260	4.1	16.6
$FeH_{0.125}$	360	6000	13.800	1860	1295	1863	1102	245	3.7	18.3
$FeH_{0.375}$	360	6000	12.824	1624	1287	1419	1237	162	6.7	8.1
Fe[a]	360	7250	13.495	1424	1182	1822	1051	162	12.4	34.6
$Fe_{60}Si_4$[b]	360	6400	13.164	1707	1353	1971	1112	191	9.1	33.4
	360	7350	13.090	1620	1403	1901	1082	168	9.8	45.1
$Fe_{56}Si_8$[b]	360	6500	12.626	1742	1397	1857	1031	172	7.9	39.5
$Fe_{60}Si_2C_2$[c]	360	6500	13.12	1597	1403	1804	1085	164	7.9	44.0
	360	6700	13.06	1562	1382	1767	1085	157	7.7	44.0
$Fe_{62}C_2$[c]	360	6400	13.36	1700	1344	1741	1057	172	5.4	31.6
$Fe_{60}C_4$[c]	360	5000	13.34	1689	1370	1913	1148	170	8.1	33.6
$Fe_{62}S_2$[c]	360	6600	13.34	1724	1293	1889	1074	199	7.6	28.4
$Fe_{60}S_4$[c]	360	6000	13.22	1706	1291	1864	1081	216	6.4	25.1
	360	6500	13.15	1635	1315	1823	1063	191	7.4	31.8
$Fe_{64}H_4$[d]	360	6500	13.491	1638	1323	1735	1106	168	5.3	28.7
$Fe_{60}Si_4H_4$[d]	360	6500	13.063	1616	1326	1755	1104	151	6.6	33.3

The errors are 3-9 K in temperature, 0.02-0.15 GPa in stress, and ~2% in C_{ij}.

[a]Martorell et al. (2013)[5]; [b]Martorell et al. (2016)[11]; [c]Li et al. (2018)[12]; [d]Wang et al. (2021)[6].

Supplementary Table 2 | The adiabatic elastic properties of Fe-H alloys under inner core conditions

	P (GPa)	T (K)	ρ (g cm^{-3})	C_{11} (GPa)	C_{12} (GPa)	C_{33} (GPa)	C_{13} (GPa)	C_{44} (GPa)	AV_P (%)	AV_S (%)
	360	0	13.751	2390	1202	2529	1107	508	5.1	10.4
	360	2000	13.654	2303	1208	2403	1116	458	4.7	10.6
	360	3000	13.552	2239	1228	2239	1114	395	3.9	12.3
$FeH_{0.25}$	360	4000	13.423	2107	1215	2116	1130	340	3.8	13.6
	360	5000	13.277	1938	1283	1899	1180	248	3.8	14.0
	330	5500	12.898	1716	1233	1681	1036	198	4.6	20.8
	360	6000	13.118	1811	1256	1762	1132	178	5.1	22.2
$FeH_{0.0625}$	360	6000	13.973	1979	1355	2010	1200	260	4.0	16.6
$FeH_{0.125}$	360	6000	13.800	1944	1379	1947	1186	245	3.6	18.3

Supplementary Discussion 2. Reversal of the fast velocity axis and change in the c/a ratio in superionic Fe-H alloys

In this section, we provide a detailed method for seismic velocity anisotropy calculation and a discussion on the relationship between seismic anisotropy and the lattice c/a ratio.

To further investigate the elastic anisotropy, we calculated the azimuthal angle-dependent velocity of superionic Fe-H alloys by solving the following Christoffel equation[13,14].

$$\rho V^2 = C_{ijkl} n_i w_j w_k n_l,\ (i, j, k, l = 1,2,3), \tag{14}$$

where n is the propagation direction and w is the polarization direction. Here, we investigated velocity anisotropy by analysing the velocity change as a function of ray angle with Earth's rotation axis. For a hexagonal system with cylindrical symmetry, we have:

$$\rho V_P^2 = C_{11} + (4C_{44} + 2C_{13} - 2C_{11})cos^2\xi + (C_{33} + C_{11} - 4C_{44} - 2C_{13})cos^4\xi, \tag{15}$$

where ρ is the density, V_P is the compressional velocity, and ξ is the angle between the ray path in the IC and Earth's rotation axis. The compressional wave velocity anisotropy AV_P and the maximum shear wave splitting anisotropy AV_S in Fe alloys (Supplementary Table 1) are calculated by:

$$AV_P = \frac{(V_P^{MAX} - V_P^{MIN}) \times 200}{V_P^{MAX} + V_P^{MIN}}, \tag{16}$$

$$AV_S = \left[\frac{(V_{S1} - V_{S2}) \times 200}{V_{S1} + V_{S2}}\right]^{MAX}. \tag{17}$$

The velocity anisotropies are also calculated by solving the Christoffel equation employing MTEX[15], as shown in Supplementary Fig. 3.

Interstitial H impurities have a significant influence on the seismic velocity anisotropy in hcp Fe. The reversal of the fast velocity direction from the c-axis to the basal plane is observed in the Fe-H alloy with increasing temperature and H content (Fig. 1). The c/a ratio is an important parameter governing the elastic anisotropy of the hcp structure[16-19]. The c/a ratio of hcp-Fe increases with temperature and approaches the

ideal value (1.633) at the temperature condition of the IC[20,21]. As a result, the elastic anisotropy of hcp-Fe under IC conditions is supposed to be weak. However, a recent experimental study suggests that the c/a ratio of hcp-Fe is substantially lower than the ideal value, indicating significant anisotropy in hcp-Fe under IC conditions[19] (Supplementary Fig. 4). We calculated the cell parameters using AIMD simulations at different temperatures and pressures under hydrostatic conditions. Our calculated c/a ratio of pure Fe at 360 GPa and 6000 K is approximately 1.61, which is consistent with previous experimental measurements, and the c/a ratio of the Fe-H alloy is larger than that of pure Fe. The c/a ratio grows further with temperature and H content, but it is still lower than the ideal value. Unlike pure Fe, the seismic anisotropy shows a trend of initially decreasing and then increasing with the c/a ratio. This effect is mainly caused by the significant decrease in C_{33} with increasing temperature. It is likely that the highly diffusive H-ions taking more unstable interstitial sites at high temperature within the basal plane are responsible for the elastic softening in C_{33} (Supplementary Fig. 5).

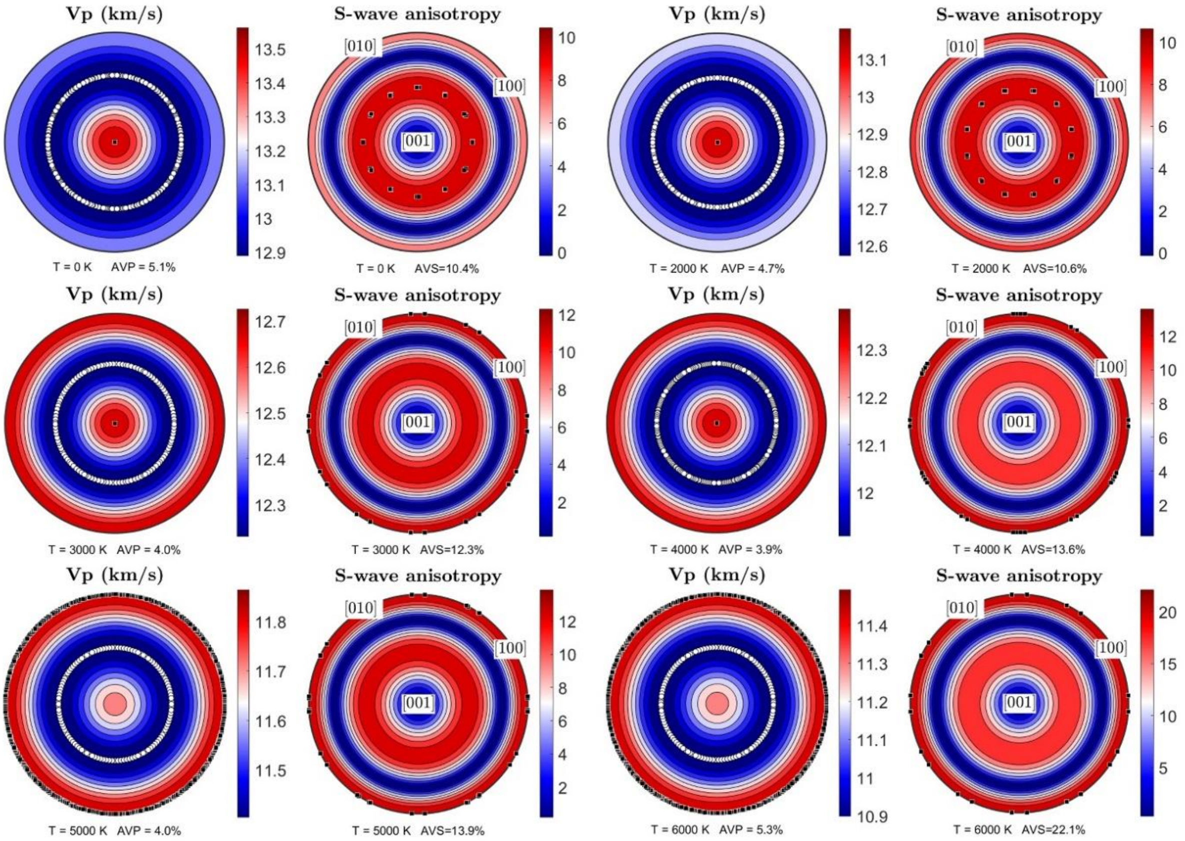

Supplementary Fig. 3 | Seismic velocity anisotropy in $FeH_{0.25}$ at 360 GPa and 0-6000 K. Black squares mark the maximum, and white circles mark the minimum. AV_P denotes the anisotropy of the P-wave velocity, while AV_S denotes the anisotropy of the S-wave velocity anisotropy (Eq. 16 and 17), which were directly obtained by solving the Christoffel equation employing MTEX[15].

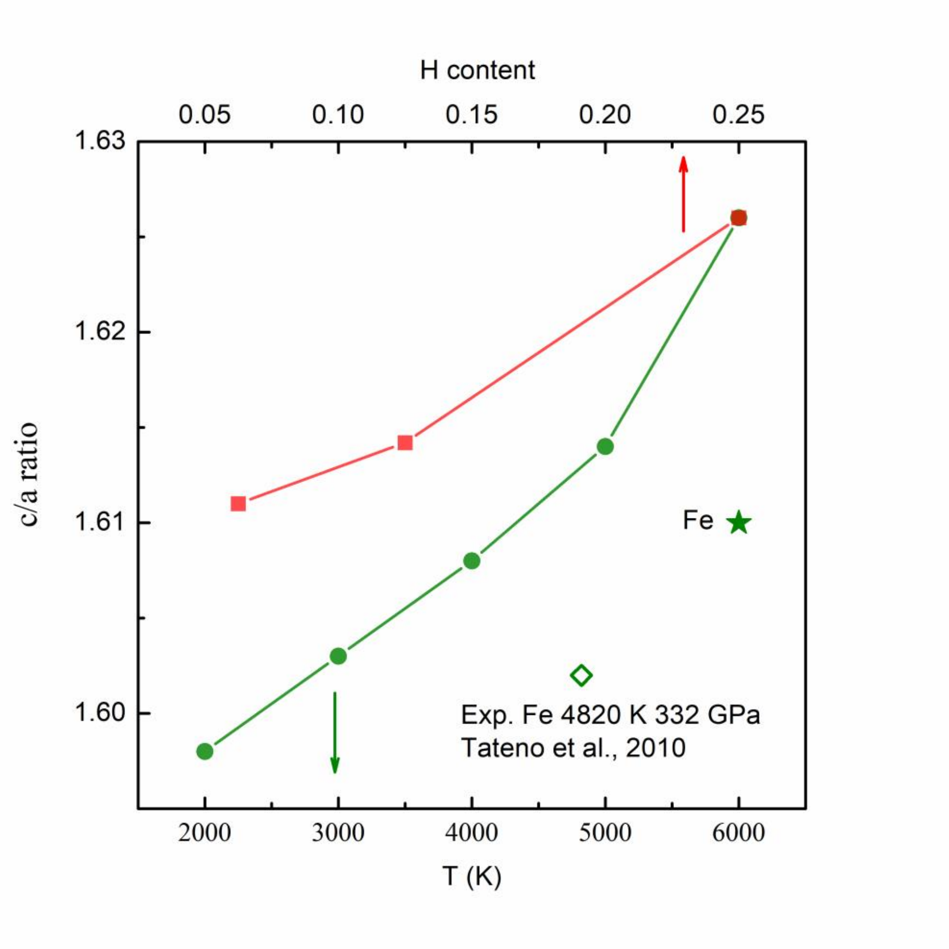

Supplementary Fig. 4. | The change in the c/a ratio as a function of temperature (green circles) and H content (red squares) in hcp-FeH$_x$ (x < 0.25) collected at 360 GPa. The calculated c/a ratio of hcp-Fe at 360 GPa and 6000 K is shown with a green start in comparison with the experimental data at 332 GPa and 4820 K (green empty diamond)[19].

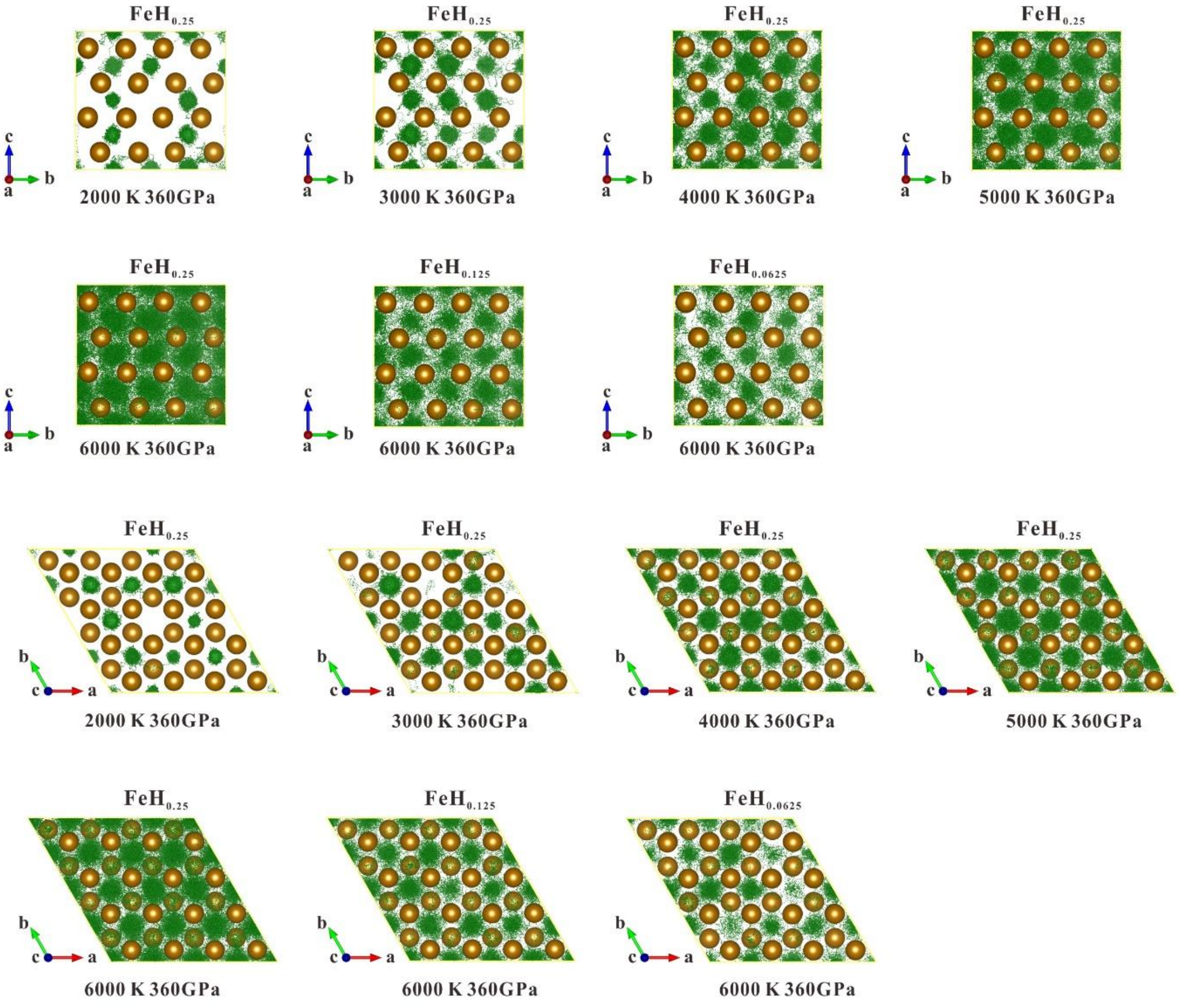

Supplementary Fig. 5 | The trajectories of H-ions in hcp-FeH_x ($x < 0.25$) alloys at 360 GPa for over 10000 steps. H-ions are represented with small green spheres, and the brown spheres display the lattice positions of Fe. The lattice is viewed along the a-axis and c-axis in the upper and lower panels, respectively.

Supplementary Discussion 3. Elastic anisotropy in Fe-H and other Fe alloys

Here, elastic anisotropies in Fe alloys are compared and discussed. Using the calculated elastic constants of previous computational studies, the compressional wave velocity anisotropies in pure Fe[5] and other Fe alloys (Fe-C, Fe-S, Fe-Si, Fe-Si-H, and Fe-Si-C)[6,11,12] are calculated and presented in Supplementary Fig. 6. In the hcp structure, the velocity anisotropies of the Fe alloy are weaker than those of pure Fe. The velocity is faster along the c-axis, which is consistent with pure Fe. This suggests that the reduction in velocity anisotropy is due to the relatively strong softening along the c-axis in the presence of light element impurities. On the other hand, superionic $FeH_{0.25}$ presents different velocity anisotropy with the fastest velocity in the basal plane.

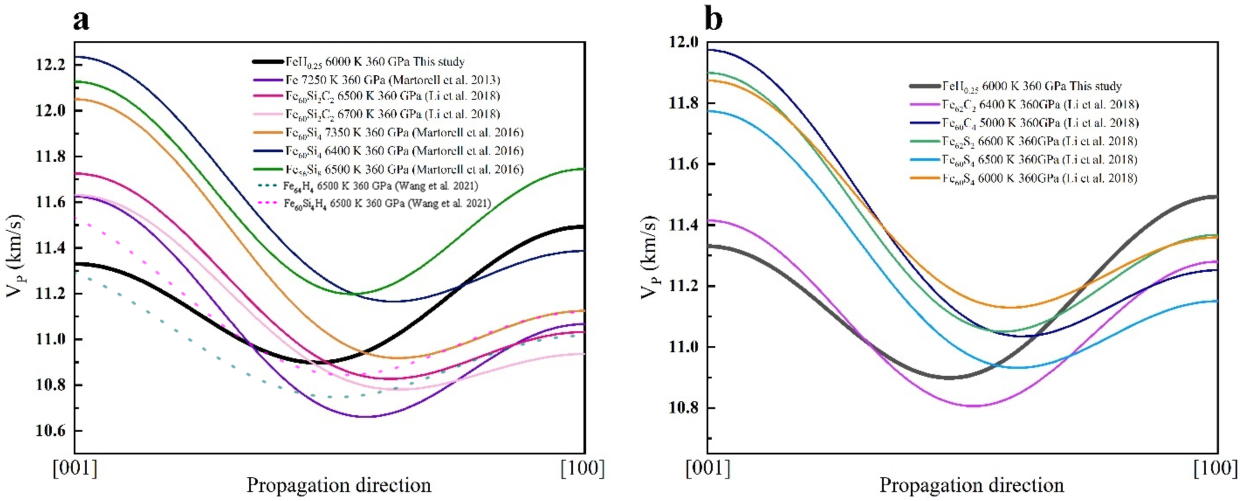

Supplementary Fig. 6 | Variation in compressional velocities (V_P) with propagation direction in superionic $FeH_{0.25}$ at 6000 K and 360 GPa in the (010) plane. (**a**) Velocities of $FeH_{0.25}$ calculated in this study compared with previously reported velocities in Fe, $Fe_{60}Si_2C_2$, $Fe_{60}Si_4$, $Fe_{56}Si_8$, $Fe_{64}H_4$, and $Fe_{60}Si_4H_4$. (**b**) Velocities of $FeH_{0.25}$ calculated in this study compared with previously reported velocities in $Fe_{62}C_2$, $Fe_{62}S_2$ and $Fe_{60}S_4$.

Supplementary Discussion 4. Comparison of Fe-H seismic anisotropy with seismological observations

In this section, the velocity anisotropies are compared with observations. IC anisotropy has been observed by body waves[22-31] and normal modes[32-34]. Based on these observations, seismic waves travelling along Earth's rotation axis are generally 2-3% faster than waves travelling along the east–west direction. With additional ultra-polar data, the anisotropy calculated by body wave residuals is better constrained to be ~2%, which is consistent with the value obtained using normal modes[31]. In considering the velocity change in $FeH_{0.25}$ with azimuthal angle, we need to deposit its basal plane parallel to Earth's rotation axis, as shown in Supplementary Fig. 7. Here, we considered two models for the configuration of $FeH_{0.25}$ orientations in the equatorial plane. The first model is the anisotropic equatorial plane (AEP) model, in which the lattice of $FeH_{0.25}$ is anisotropically oriented in the IC equatorial plane. The AEP model is an ideal model assuming that the velocity variation with ξ is consistent with the variation from the a-axis to the c-axis (Supplementary Fig. 7. AEP). This model is almost impossible in adequate sampling of all the seismic ray paths in the IC. However, the sampling should be insufficient and biased due to the inhomogeneous distribution of seismic stations. In particular, the sampling of ultra-polar paths is quite limited. In this case, constructing this model is valuable in comparison with insufficient seismic data. The second model is an isotropic equatorial plane (IEP) model with a random distribution of the $FeH_{0.25}$ lattice in the equatorial plane (IEP, Supplementary Fig. 7a). In this model, the change in the velocity as a function of angle ξ between the ray path and Earth's rotation axis is calculated by averaging the velocities of all the possible propagation direction paths corresponding to the different alignment of crystal with a rotatable c-axis in the equatorial plane (Supplementary Fig. 7b). As shown in Supplementary

Fig.7b, the crystal has a rotational degree of freedom θ in the equatorial plane. The propagation direction vector is $\boldsymbol{n}(n_1, n_2, n_3)$. Thus, we have:

$$\begin{cases} n_1 = \cos\xi \\ n_2 = \sin\xi\sin\theta \\ n_3 = \sin\xi\cos\theta \end{cases} \tag{18}$$

and we can get $V_P(\xi,\theta)$ by substituting $\boldsymbol{n}(\cos\xi, \sin\xi\sin\theta, \sin\xi\cos\theta)$ into Eq. 14 and solving the Christoffel equation. In the IEP model, we set the sampling interval of θ as 1° and $V_P(\xi)$ was calculated by:

$$V_P(\xi) = \frac{1}{360}\sum_{\theta} V_P(\xi,\theta), \theta = 1,2,\dots,360 \tag{19}$$

It can be viewed as a cylindrically averaged (averaged over θ) aggregate with a basal plane parallel to Earth's rotation axis. These two models are compared with the seismic travel time data, as discussed in the main text (Fig. 4). Here, PKIKP refers to the compressional wave path transmitted through the inner core. PKPab and PKPbc are used as reference phases traversing only the mantle and outer core. Generally, the AEP model presents the slowest angle, which fits the IMIC absolute PKIKP data better[25]. This may be due to insufficient ray path sampling of the IMIC. Alternatively, the IMIC may present a highly anisotropic equatorial plane, which is also suggested by recent observations of the coda-correlation wavefield. The equatorial anisotropy was proposed based on earthquake coda correlation, which is able to sample the deepest part of the IC[35,36]. As suggested by recent studies[37,38], earthquake coda correlation can be an efficient approach to provide additional constraints on Earth's interior structure if the real reconstructed ray paths from the coda correlation wavefield are carefully interpreted. Understanding the discrepancy between coda interferometry and ballistic observations[39,40] may further provide critical clues for understanding the IC anisotropy structure. In addition, with the increase in seismological observations and the

application of the coda correlation method, more information from the deepest part of Earth will be collected, and the mystery of the IMIC may be revealed. In particular, if the proposed equatorial anisotropy of the IMIC is confirmed with additional data, it should be critical evidence of the presence of a superionic Fe-H alloy in the IC.

We also compared the velocity anisotropy of other Fe alloys (360 GPa)[5,11,12] with the IMIC observation data[25]. In the case of other Fe alloys, the fastest direction is along the c-axis. To compare with the seismological observations, we need to place the c-axes of these Fe alloys along Earth's rotation axis, and the orientation of their lattice in the equatorial plane does not affect the velocity change with ξ. The velocity anisotropies in other Fe alloys, which roughly fit with the IMIC data, present the slowest angles from ~45-60° and significant velocity fluctuations (Supplementary Fig. 8).

The calculated velocities are also compared with the travel time residuals of PKPbc-PKIKP and PKPab-PKIKP, which are sensitive to the upper 350 km and 1100 km of the IC, respectively. However, the ray paths for these waves are different in the mantle; thus, the residuals are also affected by mantle heterogeneity. In Supplementary Fig. 9, we compared our results with the dataset after mantle and ray path corrections were reported most recently[31]. The data travelling from the South Sandwich Islands (SSI) to Alaska are shown with red circles, as the anomalous positive travel time may be caused by the presence of subducted slabs in the SSI path in the mantle[41,42]. Excluding the SSI data, the anisotropy for the bulk IC is approximately 2%, which is close to the normal model observations. The travel-time residual for $FeH_{0.25}$ was estimated by

$$\frac{\delta t}{t} = \frac{V_P - V_{P0}}{V_{P0}}, \quad (20)$$

where V_{P0} is the Voigt average velocity. As the velocity anisotropy does not show an obvious change with pressure from 330 to 360 GPa, the data calculated at 360 GPa and 6000 K were used for comparison. The calculated travel-time residual of $FeH_{0.25}$ is able to account for the IC anisotropy with faster velocity along the polar direction. We also calculated velocity anisotropies in the situation of 25%, 50%, and 75% aligned crystals with basal planes parallel to the rotation axis (Supplementary Fig. 10). In comparison with the recent dataset of seismological observations, approximately 55-70% of LPO is needed to meet the observed residuals of PKPbc-PKIKP, and ~70-80% of LPO is needed to fit the PKPab-PKIKP data. In particular, the IEP model presents milder fluctuations in the average velocity at the equatorial side, which fits the observation data better. In contrast, the other Fe alloys all present significant negative residuals.

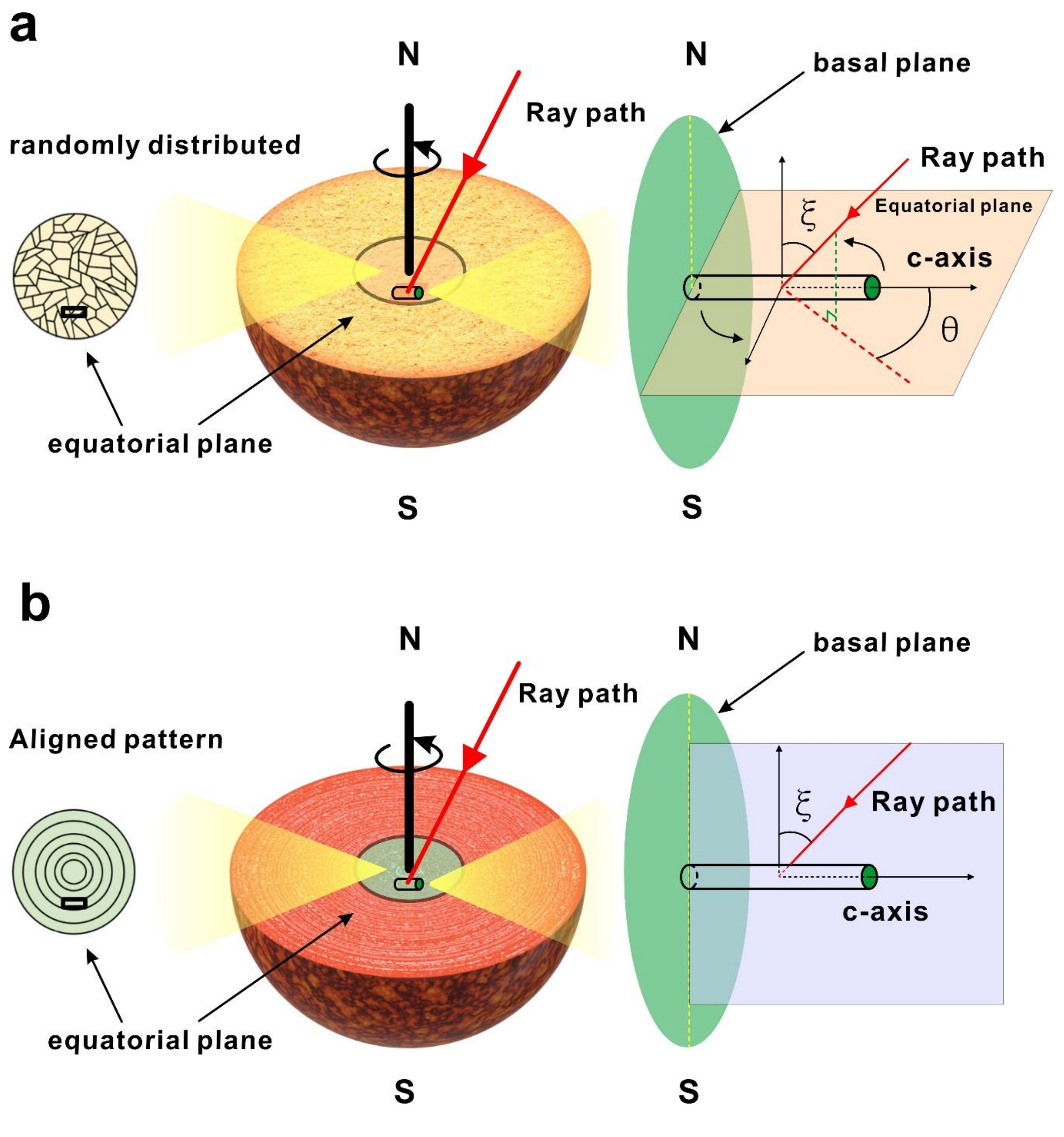

Supplementary Fig. 7 | Anisotropic Models for hcp-FeH$_{0.25}$ with the basal plane along Earth's rotation axis. (**a**) Isotropic equatorial model (IEP) model with c-axis aligned perpendicularly to the polar direction and randomly distributed in equatorial plane (left and middle panel). In the right panel, the red dashed line in equatorial plane denotes the projection line of the ray path. For a given ray-angle (ξ), the crystal has a rotational degree of freedom θ in the equatorial plane. This model is viewed as a cylindrically averaged (averaged over θ) aggregate with a

basal plane parallel to the rotation axis. (**b**) c-axis is aligned in some patterns (left and middle panel) to allow the velocity variation with ξ is consistent with the velocity change from the basal plane to the c-axis (right panel).

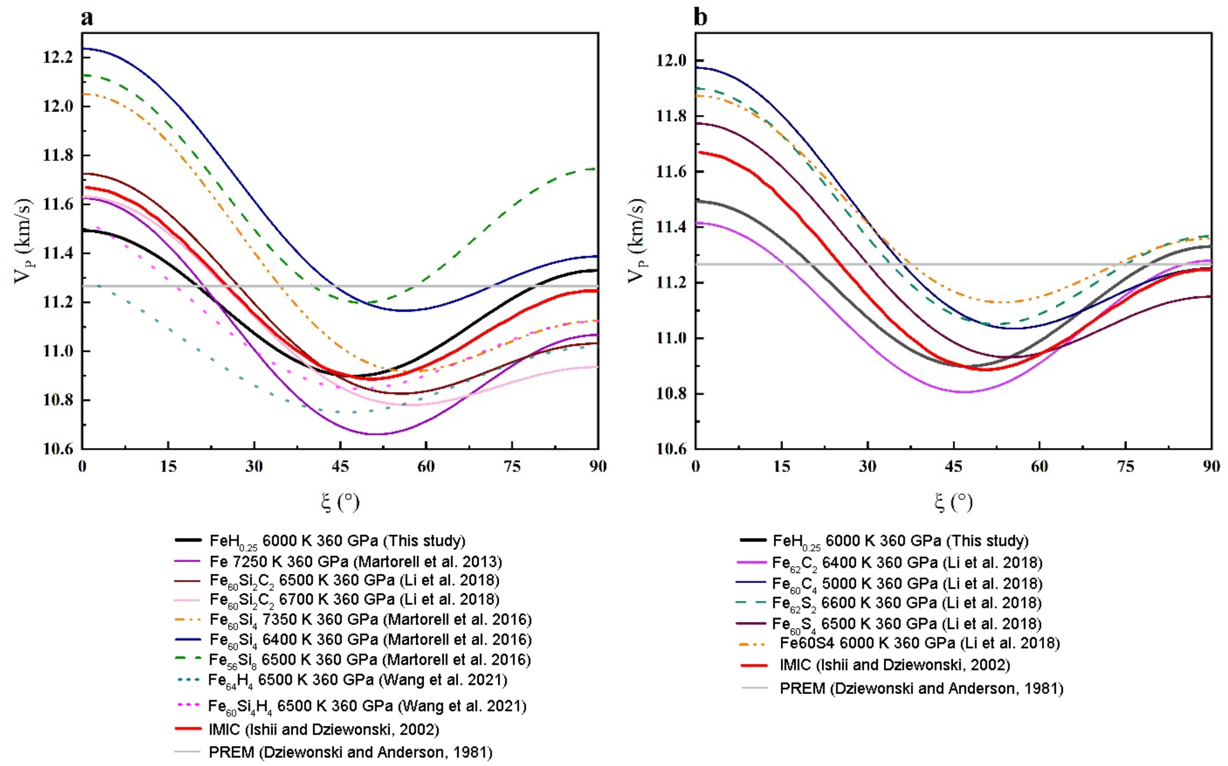

Supplementary Fig. 8 | Compressional velocity in $FeH_{0.25}$ as a function of angle *ξ* between the ray path and Earth's rotation axis. (**a**) The IMIC data fit and PREM model and previously reported Fe-Si alloys and Fe-Si-C alloys were added for comparison. (**b**) IMIC data fit and PREM model and previously reported Fe-C alloys and Fe-S alloys were plotted for comparison.

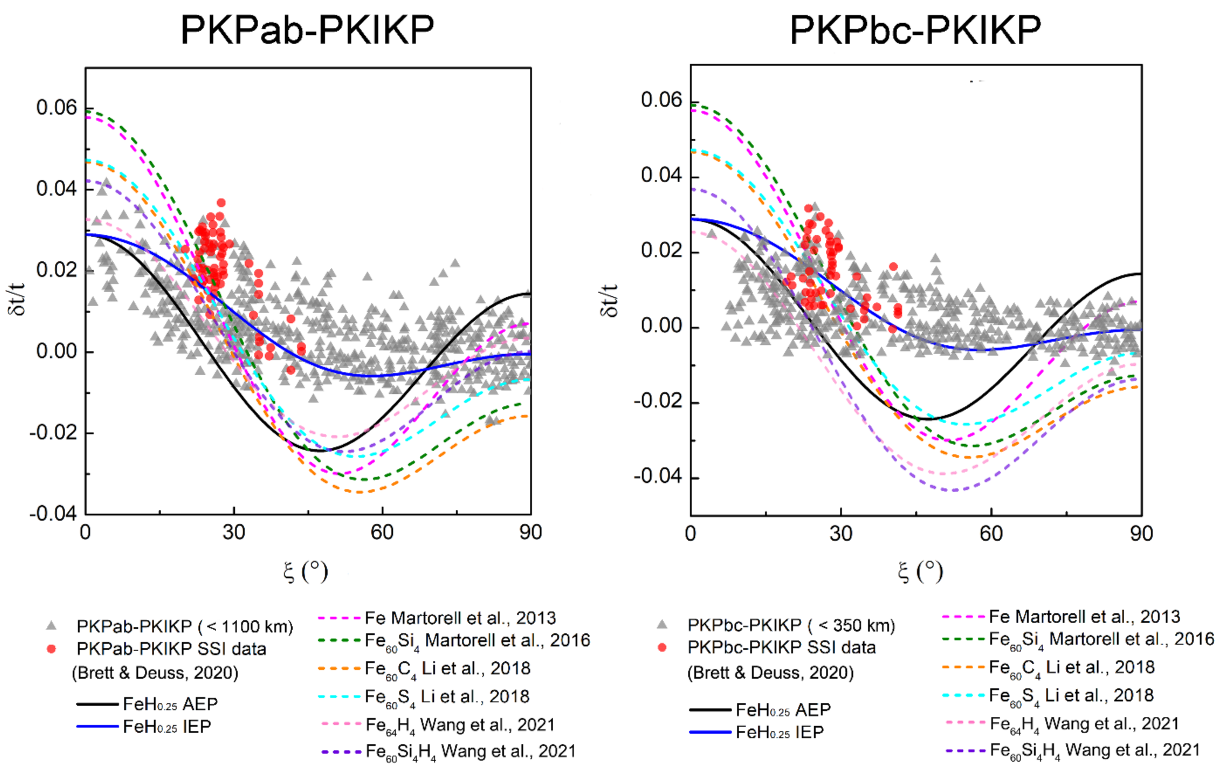

Supplementary Fig. 9 | Calculated compressional velocity anisotropy in comparison with differential travel-time residuals as a function of angle *ξ* between the ray path and Earth's rotation axis. The δt/t of $FeH_{0.25}$ and other Fe alloys are estimated by Eq. 20. The PKPab-PKIKP and PKPbc-PKIKP data are reported in a recent study with mantle and ray path corrections[31]. The data travelling from the South Sandwich Islands (SSI) to Alaska are shown with red circles.

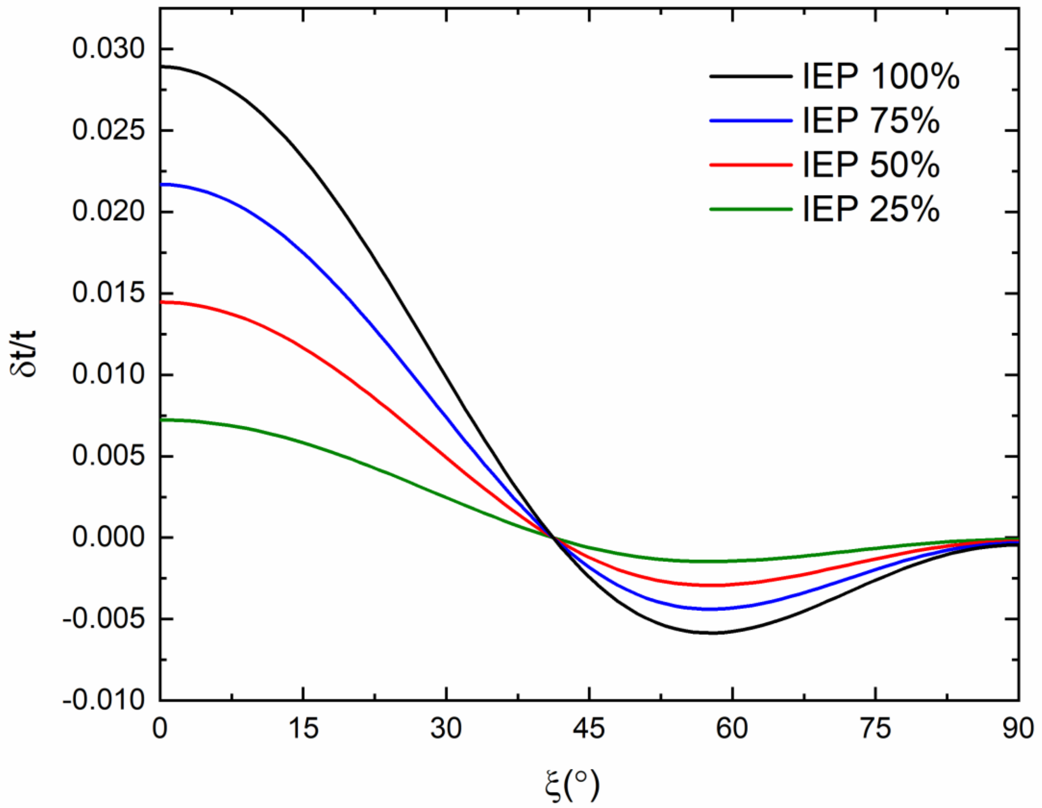

Supplementary Fig. 10 | Calculated seismic anisotropies with 25, 50, 75, and 100% LPO of hcp-$FeH_{0.25}$ with basal planes aligned with the rotation axis.

Supplementary Discussion 5. H-ion anisotropic diffusion in Fe-H alloys

In this section, we provide detailed methods and discussions for the diffusion properties of Fe-H alloys. The migration barrier energies along different migration paths are calculated using the climbing-image nudged elastic band (CINEB) method[43]. This CINEB method duplicated a series of images (7 images in our calculations) between the starting point and the end point of migrating ions to simulate the intermediate states, with the positions of the starting point and the end point fixed. The actual diffusion pathway and migration barrier energy between the starting and end points are obtained by searching for the saddle point. Only the Γ point was adopted for k-point sampling to reduce the computational cost. The convergence check indicates that a denser k-mesh does not qualitatively affect our conclusion. The cut-off energy is 600 eV. To investigate the barrier energies of H-ion migration from one site to another, larger $8 \times 4 \times 2$ and $4 \times 4 \times 4$ supercells containing 160 atoms were used to calculate the barrier energies along the a-axis and c-axis, respectively. The cell parameters and volumes of hcp-$FeH_{0.25}$ at 2000-6000 K and 360 GPa are adopted from AIMD simulations, and both the volumes and c/a ratios increase with temperature (Supplementary Fig. 4).

The stable interstitial site is the octahedral site (O), and the tetrahedral site (T) is metastable. We considered three diffusion paths for H-ions. As shown in Fig. 2a, path 1 and path 2 are in the basal plane, and the direct migration from O-O in the basal plane is much less stable than the indirect migration path O-T-O. In the direction along the c-axis, the direct migration from O-O (path 3) presents the lowest barrier energy. The saddle points (S) for these migration paths are at the center of the octahedral planes, where the H-ion has the strongest interaction with the Fe lattice. The barrier energies along the c-axis and in the basal plane are different due to the nonideal c/a ratio. In

$FeH_{0.25}$ at 360 GPa and 6000 K, the c/a ratio is 1.626, which is smaller than the ideal value of 1.633. As the distance of nearby Fe atoms along the c-axis is shorter than the distance in the basal plane, H-ion migration in the basal plane has a stronger interaction with the Fe sublattice. Thus, the c/a ratio is a critical parameter governing the anisotropic diffusion behavior of interstitial impurities in hcp-Fe alloys, and diffusion in the basal plane can be favorable when the c/a ratio is larger than the ideal value. In the case of hcp-$FeH_{0.25}$, the c/a ratios are lower than the ideal value under IC conditions, and H-ion diffusion along the c-axis is more favorable. From 2000 to 6000 K, the barrier energies for the three paths mostly decrease with temperature, and the barrier energy along the c-axis is the lowest (Fig. 2c).

We also used AIMD simulations to study the diffusion anisotropy in hcp-Fe-H alloys. In the AIMD simulations, the diffusion coefficient is defined as:

$$D = \lim_{0\to\infty} \left[\frac{1}{2dt}\langle[\vec{r}(t)]^2\rangle\right], \tag{21}$$

where d is the dimension of the lattice on which ion hopping takes place. Mean square displacement (MSD)

$$\langle[\vec{r}(t)]^2\rangle = \frac{1}{N}\sum_{i=1}^{N}\langle[\vec{r_i}(t+t_0) - \vec{r_i}(t_0)]^2\rangle, \tag{22}$$

is averaged over all H-ions, $\vec{r_i}(t)$ is the displacement of the ith H-ion at time t, and N is the total number of H-ions in the system.

The H-ion diffusion coefficients along different directions in $FeH_{0.25}$ at 2000-6000 K and 360 GPa are calculated and plotted as a function of reciprocal temperature (Fig. 2d). The values are fitted with an Arrhenius equation:

$$D = \mathrm{A}\exp\left(-\frac{\Delta \mathrm{H}}{\mathrm{kT}}\right), \tag{23}$$

where ΔH is the activation enthalpy, A is a preexponential factor, k is the Boltzmann constant, and

T is the temperature. Based on Eq. 21, ΔH along different paths were calculated, and the activation enthalpy along the c-axis was the lowest (Fig. 4d). This result is consistent with the migration barrier energy calculated using the CINEB method, suggesting that H-ion diffusion along the c-axis is the energetically most favorable path in $FeH_{0.25}$. The diffusion coefficient of defects in the lattice can be described by the following equation:

$$D = \frac{Z}{6} F l^2 \upsilon e^{-\frac{\Delta H - T\Delta S}{kT}}, \quad (24)$$

where Z is the number of equivalent migration paths, F is the correlation factor, l is the migration distance, υ is the attempt frequency, ΔH and ΔS are the migration enthalpy and entropy, k is Boltzmann's constant, and T is the temperature. As shown in Supplementary Fig. 11, there are two equivalent migration paths for H ions along the a-axis of the hcp-Fe lattice, while there is only one migration path along the c-axis. Therefore, the H-ion diffusion coefficients along the a-axis and c-axis are almost identical at high temperatures (Fig. 4d) although the migration barrier energy along the c-axis is higher.

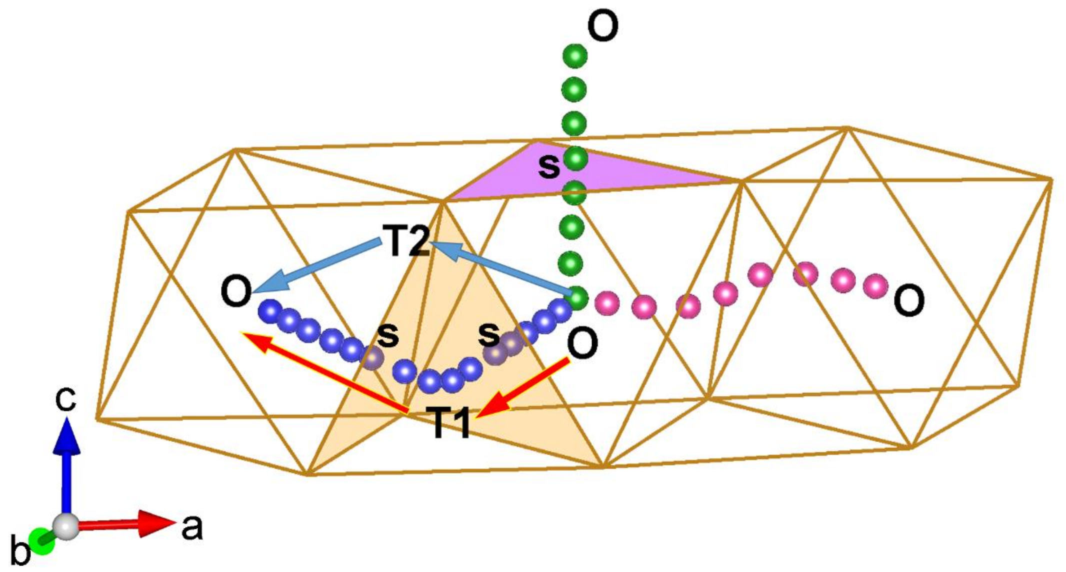

Supplementary Fig. 11. The equivalent migration paths along the a-axis. The equivalent migration paths, O-T1-O and O-T2-O, are shown with blue and red arrows. O, T, and S represent the octahedral site, tetrahedral site, and saddle point of the migration path, respectively.

Supplementary Discussion 6. H-ion transportation in an external electric field

External electric fields alter ionic transportation behavior in superionic ice[44]. Considering the presence of an electric field in Earth's inner core, it is worth studying the effect of an external electric field on H-ion diffusion in superionic Fe-H alloys. MD simulations based on neural network potential (NNP) with a large supercell and long simulation time are used to enhance computational efficiency and accuracy. Thus, an NNP of $FeH_{0.25}$ at 360 GPa and 6000 K is developed based on our AIMD dataset using the DeePMD-kit package[45]. The NNP and DFT computed energies and forces are compared in Supplementary Fig. 12, and the root mean square errors are 4 meV atom^{-1} for the energies, 256 meV Å^{-1} for the forces, and 24 meV atom^{-1} for the virial stresses on the testing set at 360 GPa and 6000 K. Comparisons between the pair distribution function computed by the NNP-based MD simulation and AIMD show good consistency (Supplementary Fig. 13).

We conducted nonequilibrium molecular dynamics simulations using the LAMMPS software package[46,47] with the well-trained NNP. A larger supercell containing 640 atoms was used for the simulation, and the temperature was controlled by the Nosé–Hoover thermostat. External static electric fields with strengths of 0.1 and 1.0 V Å^{-1} were set along the directions of the a-axis and c-axis of the hcp-$FeH_{0.25}$ lattice and run for 1000 ps in a canonical ensemble at 360 GPa and 6000 K.

The incorporation of an electric field into MD simulations can be achieved by adding a force $\mathbf{F}=q\mathbf{E}$[48] with a charge of q. The atomic charges of Fe and H were set to +0.075 e and -0.3 e according to the Bader charge calculation results (Table 3). The total energy in MD simulations was calculated by counting the contributions from potential energy, kinetic energy and electric-field-induced

energy change.

The MSDs of the H-ion along the a-axis and c-axis were calculated and are shown in Supplementary Fig. 14. The MSD changes with the applied electric field, and diffusion along the c-axis is more favorable when the electric field points to the c-axis. However, the electric field along the a-axis does not significantly increase H-ion diffusion along this direction. In particular, when the field intensity is 0.1 V $Å^{-1}$, H-ion diffusion along the c-axis is still preferred (Supplementary Fig. 14). The calculated diffusion coefficients under different external electric fields are shown in Fig. 3a. In addition, we compared the internal energies under the electric field along the a-axis and c-axis. The energy differences ($\Delta E = E_{c\text{-}axis}\text{-}E_{a\text{-}axis}$) increase with simulation time under electric fields of 0.1 and 1.0 V $Å^{-1}$, indicating that the alignment of the c-axis parallel to the external electric field direction is energetically more favorable. This provides robust evidence of the anisotropic H-ion transportation property in the hcp-FeH alloy, especially in the presence of external electric fields.

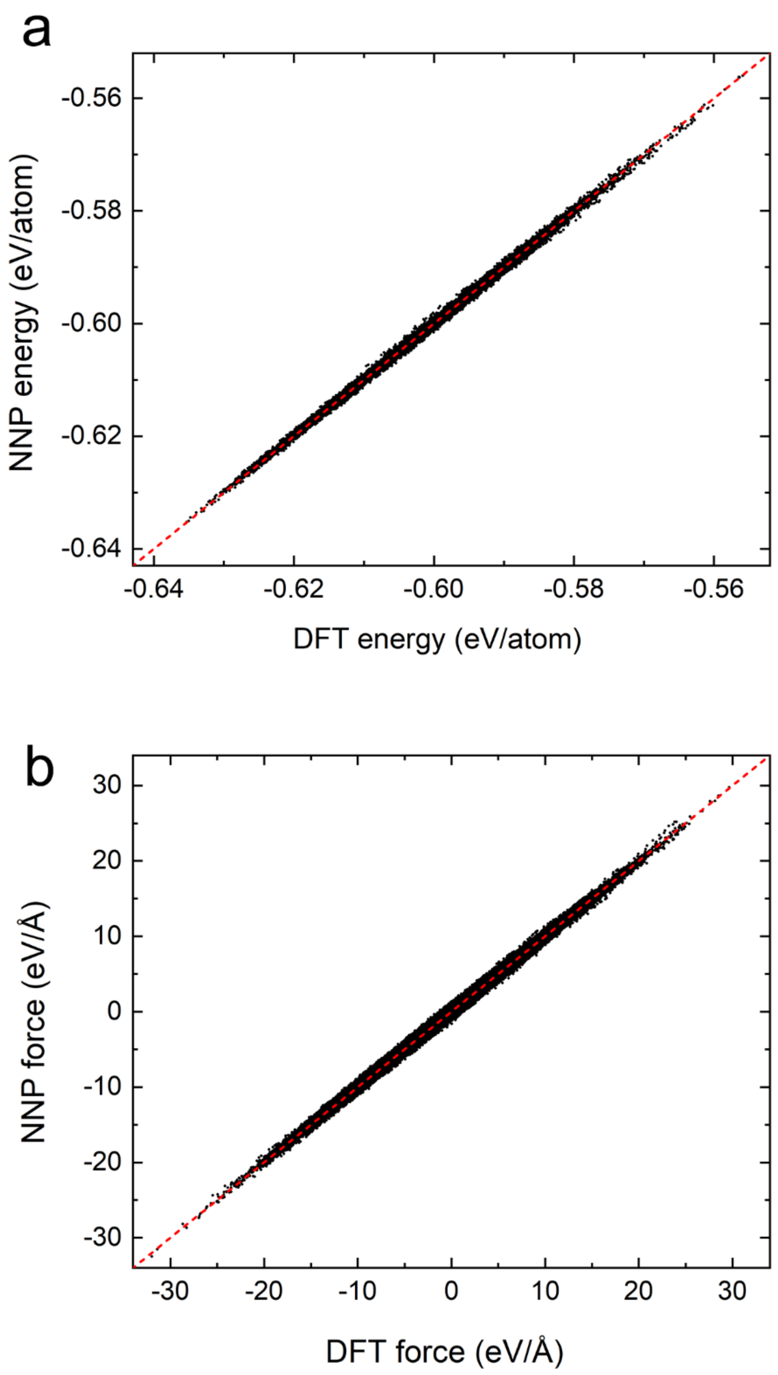

Supplementary Fig. 12 | Scatter plot of the NNP energies and forces. Comparison of the energies and forces calculated using DFT (a) and NNP (b) for a dataset of 20000 configurations.

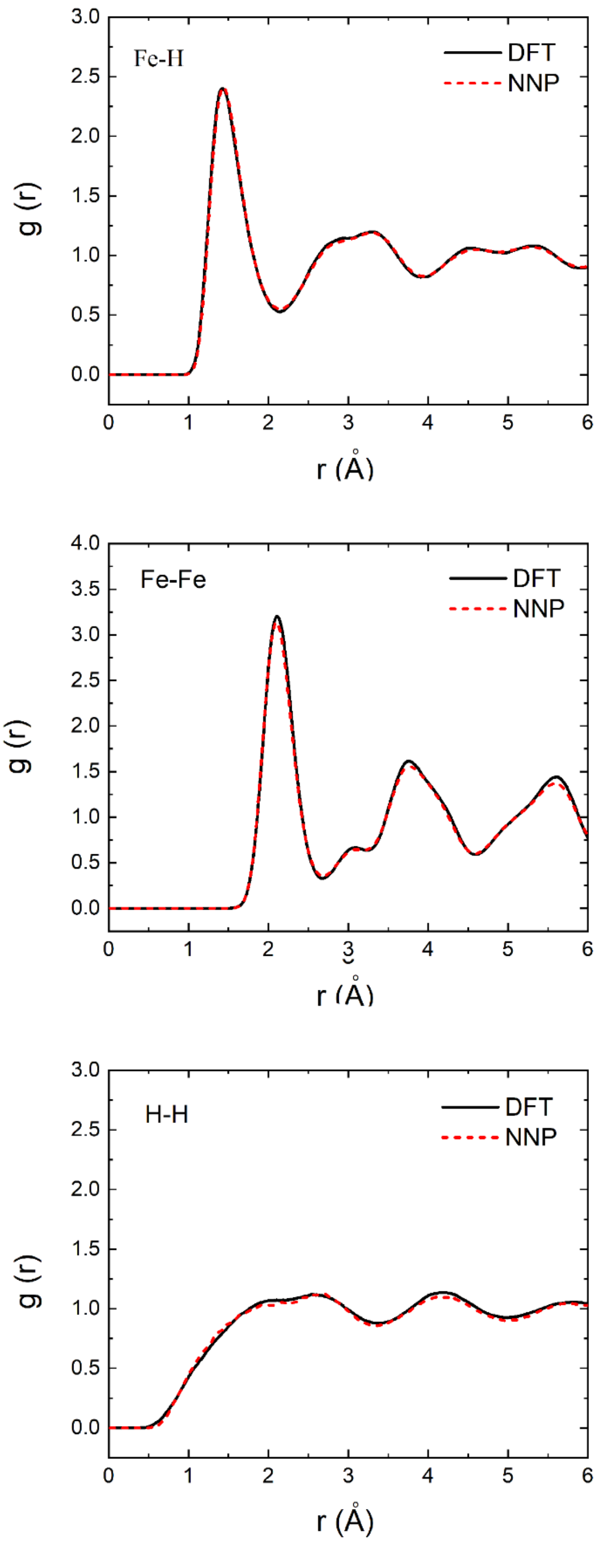

Supplementary Fig. 13 | Comparison of partial radial distribution functions (RDFs). The RDFs of Fe-H, Fe-Fe, and H-H are calculated using AIMD (black) and NNP-based MD (red) methods at 360 GPa and 6000 K.

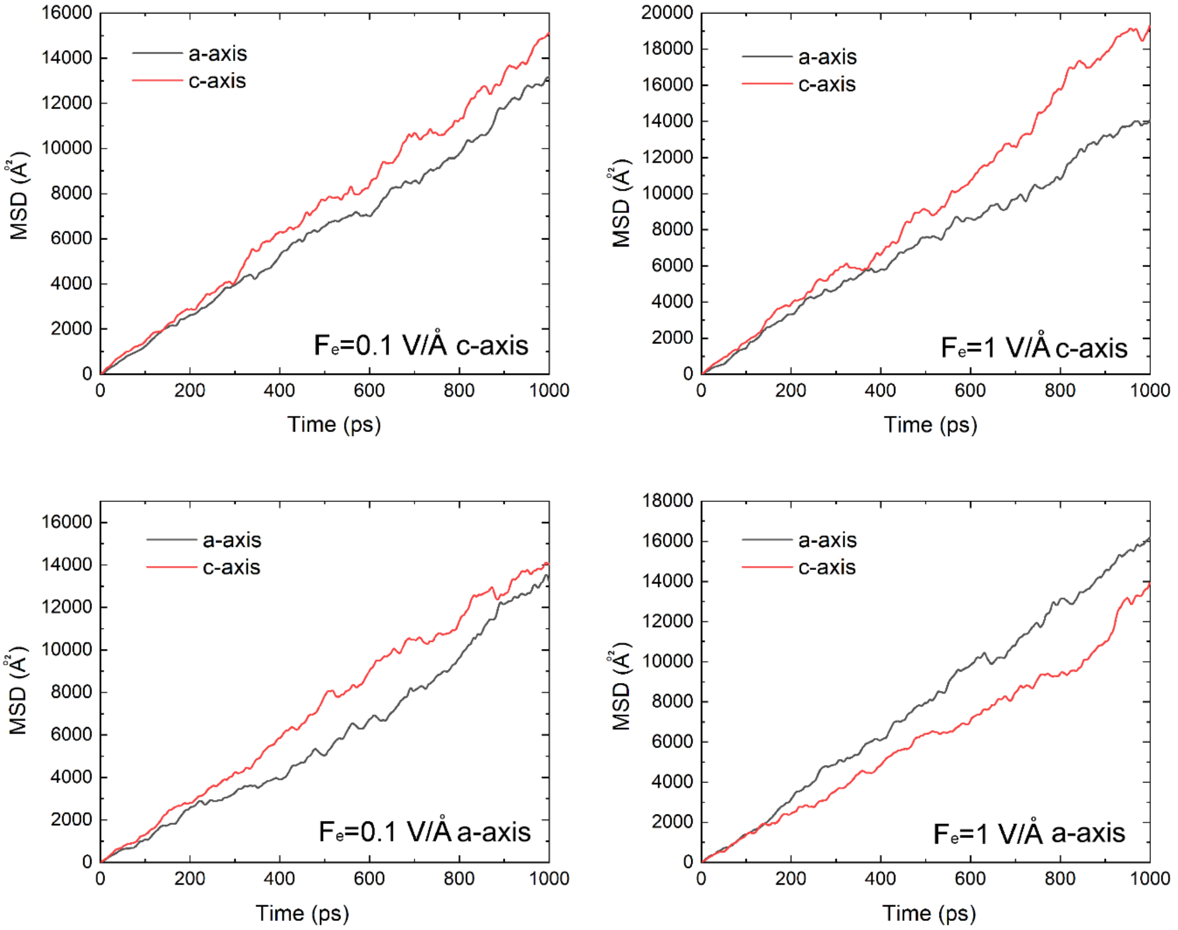

Supplementary Fig. 14 | The MSDs along the a-axis and c-axis in superionic $FeH_{0.25}$ at 360 GPa and 6000 K in the presence of external static electric fields (F_e). The field directions and intensities are labelled.

Supplementary Discussion 7. Bader charge analysis of Fe alloys

To simulate the anisotropic diffusion behavior of H-ions under an external electric field, we calculate the charge distribution first. To estimate the charge of interstitial impurities (C, H, and O) in Fe alloys, we performed Bader charge analysis[49] on the electronic charge densities of $FeH_{0.25}$, $FeC_{0.0625}$, and $FeO_{0.0625}$. The structures were obtained from our previous study[1]. The results are shown in Supplementary Table S3, and the net charges for the H, C, and O ions are -0.306, -0.803, and -0.951 e, respectively. The negative charge suggests electron transfer from Fe to interstitial H, C, and O atoms in these alloys at 360 GPa. The calculated charge was adopted for the MD simulations under electric fields.

Supplementary Table 3. Bader net charge of the atoms for $FeH_{0.25}$, $FeC_{0.0625}$, and $FeO_{0.0625}$ at 360 GPa

$FeH_{0.25}$		$FeC_{0.0625}$		$FeO_{0.0625}$	
Atoms	charge (e)	Atoms	charge (e)	Atoms	charge (e)
H1	-0.306	C1	-0.803	O1	-0.951
H2	-0.306	Fe1	0.013	Fe1	0.009
Fe1	0.094	Fe2	0.011	Fe2	0.007
Fe2	0.059	Fe3	-0.004	Fe3	0.0004
Fe3	0.059	Fe4	0.011	Fe4	0.009
Fe4	0.094	Fe5	0.117	Fe5	0.168
Fe5	0.094	Fe6	0.113	Fe6	0.161
Fe6	0.059	Fe7	0.029	Fe7	-0.035
Fe7	0.059	Fe8	0.111	Fe8	0.157
Fe8	0.094	Fe9	0.119	Fe9	0.165
		Fe10	0.029	Fe10	-0.035
		Fe12	0.113	Fe12	0.161
		Fe11	0.108	Fe11	0.159
		Fe13	0.012	Fe13	0.009
		Fe14	-0.004	Fe14	0.0004

	Fe15	0.011	Fe15	0.00699
	Fe16	0.013	Fe16	0.009

Supplementary References

1. He, Y., Sun, S., Kim, D. Y., Li, H., Mao, H.-k. Superionic hcp-Fe alloys and their seismic velocities in Earth's inner core. *Nature* **602**, 258-262 (2022).
2. Sun, S., He, Y., Kim, D. Y. & Li, H. Anomalous elastic properties of superionic ice. *Phy. Rev. B* **102**, 104108 (2020).
3. He, Y., Sun, S. & Li, H. Ab initio molecular dynamics investigation of the elastic properties of superionic Li_2O under high temperature and pressure. *Phy. Rev. B* **103**, 174105 (2021).
4. Flyvbjerg, H. & Petersen, H. G. Error estimates on averages of correlated data. *J. Chem. Phys.* **91**, 461-466 (1989).
5. Martorell, B., Vocadlo, L., Brodholt, J. & Wood, I. G. Strong premelting effect in the elastic properties of hcp-Fe under inner-core conditions. *Science* **342**, 466-468 (2013).
6. Wang, W. et al. Strong shear softening induced by superionic hydrogen in Earth's inner core. *Earth and Planetary Sci. Lett.* **568**, 117014 (2021).
7. Vočadlo, L. Ab initio calculations of the elasticity of iron and iron alloys at inner core conditions: Evidence for a partially molten inner core? *Earth and Planetary Sci. Lett.* **254**, 227-232 (2007).
8. Vočadlo, L., Alfè, D., Gillan, M. J. & Price, G. D. The properties of iron under core conditions from first principles calculations. *Phys. Earth Planet. Inter.* **140**, 101-125 (2003).
9. Simmons, G. & Wang, H. Single Crystal Elastic Constants and Calculated Aggregate Properties: A Handbook (MIT Press, Cambridge, MA, 1971).
10. Dziewonski, A. M. & Anderson, D. L. Preliminary Reference Earth Model. *Physics of the Earth and Planetary Interiors* **25**, 297-356 (1981).
11. Martorell, B., Wood, I. G., Brodholt, J. & Vočadlo, L. The elastic properties of hcp-$Fe_{1-x}Si_x$ at Earth's inner-core conditions. *Earth and Planetary Sci. Lett.* **451**, 89-96 (2016).
12. Li, Y., Vočadlo, L. & Brodholt, J. P. The elastic properties of hcp-Fe alloys under the conditions of the Earth's inner core. *Earth and Planetary Sci. Lett.* **493**, 118-127 (2018).
13. Anderson, D. Theory of the Earth (Blackwell Scientific, Boston, (1989)
14. Karki, B., Stixrude, L., Clark, S., Warren, M., Ackland, G. & Crain, J. Structure and elasticity of MgO at high pressure. *Am. Mineral.* **82**, 51 (1997).
15. Mainprice, D., Hielscher, R. & Schaeben, H. Calculating anisotropic physical properties from texture data using the MTEX open-source package. Geological Society, London, Special Publications **360**, 175-192 (2011).
16. Sha, X. & Cohen, R. E. Elastic isotropy of ε-Fe under Earth's core conditions. *Geophys. Res. Lett.* **37**, L10302 (2010).
17. Vočadlo, L., Dobson, D. P. & Wood, I. G. Ab initio calculations of the elasticity of hcp-Fe as a function of temperature at inner-core pressure. *Earth Planet. Sci. Lett.* **288**, 534–38 (2009).
18. Mattesini, M. et al., Hemispherical anisotropic patterns of the Earth's inner core. *Proc. Natl. Acad. Sci. USA* **107**, 9507-9512 (2010).
19. Tateno, S., Hirose, K., Ohishi, Y. & Tatsumi, Y. The Structure of Iron in Earth's Inner Core. *Science* **330**, 359-361 (2010).
20. Belonoshko, A. B., Ahuja, R. & Johansson, B. Stability of the body-centred-cubic phase of iron in the Earth's inner core. *Nature* **424**, 1032-1034 (2003).
21. Gannarelli, C. M. S., Alfe, D. & Gillan, M. J. The axial ratio of hcp iron at the conditions of the Earth's inner core. *Phys. Earth Planet. Inter.* **152**, 67-77 (2005).

22. Poupinet, G., Pillet, R. & Souriau, A. Possible heterogeneity of the Earth's core deduced from PKIKP travel times. *Nature* **305**, 204-206 (1983).
23. Morelli, A., Dziewonski, A.M. & Woodhouse, J.H. Anisotropy of the inner core inferred from PKIKP travel times, *Geophys. Res. Lett.* **13**, 1545–1548 (1986).
24. Creager, K.C. Anisotropy of the inner core from differential travel times of the phases PKP and PKIKP. *Nature* **356**, 309-314 (1992).
25. Ishii, M. & Dziewonski, A.M. The innermost inner core of the Earth: evidence for a change in anisotropic behavior at the radius of about 300 km, *Proc. Natl. Acad. Sci. USA* **99**, 14026–14030 (2002).
26. Niu, F. & Chen, Q.F. Seismic evidence for distinct anisotropy in the innermost inner core. *Nat. Geosci.* **1**, 692–696.
27. Niu, F. & Wen, L. Hemispherical variations in seismic velocity at the top of Earth's inner core. *Nature* **410**, 1081–1084 (2001).
28. Song, X., Helmberger, D. V. Depth dependence of anisotropy of Earth's inner core. *J. Geophys. Res.* **100**, 9805–9816 (1995).
29. Irving, J. C. E. & Deuss, A. Hemispherical structure in inner core velocity anisotropy. *J. Geophys. Res.* **116**, B04307 (2011).
30. Lythgoe, K. H., Deuss, A., Rudge, J. F. & Neufeld, J. A. Earth's inner core: Innermost inner core or hemispherical variations? *Earth Planet. Sci. Lett.* **385**, 181–189 (2014).
31. Brett, H. & Deuss, A. Inner core anisotropy measured using new ultra-polar PKIKP paths. *Geophys. J. Int.* **223**, 1230-1246 (2020).
32. Woodhouse, J.H., Giardini, D. & Li, X.-D. Evidence for inner core anisotropy from free oscillations. *Geophys. Res. Lett.* **13**, 1549–1552 (1986).
33. Deuss, A., Irving, J.C.E. & Woodhouse, J.H. Regional variation of inner core anisotropy from seismic normal mode observations. *Science* **328**, 1018–1020 (2010).
34. Irving, J. C. E., Deuss, A. & Woodhouse, J. H. Normal mode coupling due to hemispherical anisotropic structure in Earth's inner core. *Geophys. J. Int.* **178**, 962–75 (2009).
35. Wang, T., Song, X. & Xia, H. H. Equatorial anisotropy in the inner part of Earth's inner core from autocorrelation of earthquake coda. Nat. Geosci. **8**, 224-227 (2015).
36. Wang, T. & Song, X. Support for equatorial anisotropy of Earth's inner-inner core from sesmic interferometry at low latitudes. *Phys. Earth Planet. Inter.* **276**, 247-257 (2018).
37. Tkalčić, H., Phạm, T.-S. & Wang, S. The Earth's coda correlation wavefeld: Rise of the new paradigm and recent advances. *Earth-Sci. Rev.* **208**, 103285 (2020).
38. Wang, S., & Tkalčić, H. Seismic event coda-correlation: Toward global coda-correlation tomography. *J. Geophys. Res.: Solid Earth* **125**, e2019JB018848 (2020).
39. Romanowicz, B., Cao, A., Godwal, B., Wenk, R., Ventosa, S. & Jeanloz, R. Seismic anisotropy in the Earth's innermost inner core: testing structural models against mineral physics predictions. *Geophys. Res. Lett.* **43**, 93–100. (2016).
40. Frost, D.A. & Romanowicz, B. On the orientation of the fast and slow directions of anisotropy in the deep inner core. *Phys. Earth Planet. Inter.* **286**, 101–110 (2019).
41. Tkalčić, H. Large variations in travel times of mantle-sensitive seis-mic waves from the South Sandwich Islands: is the Earth's inner core a conglomerate of anisotropic domains? *Geophys. Res. Lett.* **37**, L14312 (2010).
42. Frost, D.A., Romanowicz, B. & Roecker, S., 2020. Upper mantle slab under Alaska: contribution to anomalous core-phase observations on south-Sandwich to Alaska paths. *Phys. Earth planet. Inter.* **299**,

106427 (2020).
43. Henkelman, G., Uberuaga, B. P. & Jonsson, H. A climbing image nudged elastic band method for finding saddle points and minimum energy paths. *J. Chem. Phys.* **113**, 9901–9904 (2000).
44. Futera, Z., Tse, J. S. & English, N. J. Possibility of realizing superionic ice VII in external electric fields of planetary bodies. *Sci. Adv.* **6**, eaaz2915 (2020).
45. Wang, H., Zhang, L., Han, J. & E, W. DeePMD-kit: A deep learning package for many-body potential energy representation and molecular dynamics. *Comput. Phys. Commun.* **228**, 178–184 (2018).
46. Plimpton, S. Fast Parallel Algorithms for Short-Range Molecular Dynamics. J. Comput. Phys. *117*, 1-19 (1995).
47. English, N.J. Molecular Simulation of External Electric Fields on the Crystal State: A Perspective. *Crystals* **11**, 1405 (2021).
48. Plimpton, S. LAMMPS-large-scale atomic/molecular massively parallel simulator. Sandia Natl. Lab. (2007).
49. Bader, R. F. W. Atoms in Molecules: A Quantum Theory. Oxford University Press, Oxford, UK, (1990).
